# [1-(2-Oxidobenzyl­idene)-4-phenyl­thio­semicarbazidato-κ^3^ *O*,*N* ^1^,*S*](pyridine-κ*N*)copper(II)

**DOI:** 10.1107/S1600536810017381

**Published:** 2010-05-19

**Authors:** Vladimir V. Bon, Svitlana I. Orysyk, Vasily I. Pekhnyo

**Affiliations:** a Institute of General and Inorganic Chemistry, NAS Ukraine, Kyiv, prosp. Palladina 32/34, 03680 Ukraine

## Abstract

In the structure of the title compound, [Cu(C_14_H_11_N_3_OS)(C_5_H_5_N)], the Cu^II^ atom exhibits a slightly distorted square-planar CuN_2_OS coordination polyhedron consisting of a phenyl O, an azomethine N and a thio­amide S atom from the tridentate thio­semicarbazonate dianion, and the N atom of a pyridine mol­ecule. The thio­semicarbazonate ligand exists in the thiol tautomeric form as an *E* isomer. Rotational disorder of the pyridine and phenyl rings in a 1:1 ratio of the respective components is observed. An extensive network of weak N—H⋯S, C—H⋯O, C—H⋯N and C—H⋯S hydrogen-bonding inter­actions consolidates the structure.

## Related literature

For general background to thio­semicarbazonates, see: Garoufilis *et al.* (2009 ▶); Stanojkovic *et al.* (2010 ▶); Kaur *et al.* (2007 ▶). For related structures, see: John *et al.* (2002 ▶); Naik *et al.* (2003 ▶); Cao *et al.* (2007 ▶); Seena & Kurup (2008 ▶).
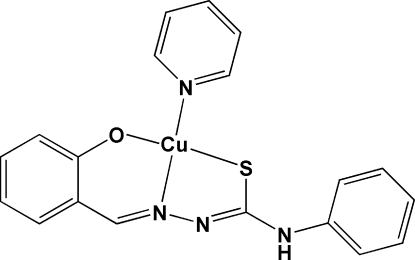


## Experimental

### 

#### Crystal data

[Cu(C_14_H_11_N_3_OS)(C_5_H_5_N)]
*M* *_r_* = 411.96
Monoclinic, 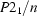

*a* = 18.2958 (17) Å
*b* = 4.5610 (5) Å
*c* = 20.473 (2) Å
β = 93.602 (7)°
*V* = 1705.1 (3) Å^3^
*Z* = 4
Mo *K*α radiation
μ = 1.42 mm^−1^
*T* = 173 K
0.50 × 0.06 × 0.05 mm

#### Data collection

Bruker APEXII CCD diffractometer
Absorption correction: multi-scan (*SADABS*; Bruker, 2005 ▶) *T*_min_ = 0.540, *T*_max_ = 0.938
19901 measured reflections
3493 independent reflections
2522 reflections with *I* > 2σ(*I*)
*R*_int_ = 0.078

#### Refinement

*R*[*F* ^2^ > 2σ(*F* ^2^)] = 0.038
*wR*(*F* ^2^) = 0.080
*S* = 1.02
3493 reflections
311 parameters
H atoms treated by a mixture of independent and constrained refinement
Δρ_max_ = 0.33 e Å^−3^
Δρ_min_ = −0.45 e Å^−3^

### 

Data collection: *APEX2* (Bruker, 2005 ▶); cell refinement: *SAINT* (Bruker, 2005 ▶); data reduction: *SAINT*; program(s) used to solve structure: *SHELXS97* (Sheldrick, 2008 ▶); program(s) used to refine structure: *SHELXL97* (Sheldrick, 2008 ▶); molecular graphics: *SHELXTL* (Sheldrick, 2008 ▶); software used to prepare material for publication: *publCIF* (Westrip, 2010 ▶).

## Supplementary Material

Crystal structure: contains datablocks I, global. DOI: 10.1107/S1600536810017381/wm2343sup1.cif

Structure factors: contains datablocks I. DOI: 10.1107/S1600536810017381/wm2343Isup2.hkl

Additional supplementary materials:  crystallographic information; 3D view; checkCIF report

## Figures and Tables

**Table 1:** Selected bond lengths (Å)

Cu1—O1	1.9126 (19)
Cu1—N1	1.926 (2)
Cu1—N4	2.010 (2)
Cu1—S1	2.2626 (8)

**Table 2:** Hydrogen-bond geometry (Å, °)

*D*—H⋯*A*	*D*—H	H⋯*A*	*D*⋯*A*	*D*—H⋯*A*
N3—H3*N*⋯S1^i^	0.74 (3)	2.90 (3)	3.593 (3)	157 (3)
C10*A*—H10*A*⋯N2	0.95	2.28	2.852 (7)	118
C10*B*—H10*B*⋯N2	0.95	2.43	2.936 (7)	113
C14*B*—H14*B*⋯S1^i^	0.95	2.81	3.679 (6)	152
C15*B*—H15*B*⋯O1^ii^	0.95	2.40	3.339 (7)	169
C16*A*—H16*A*⋯O1^iii^	0.95	2.53	3.371 (7)	148

Symmetry codes: (i) 
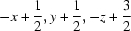
; (ii) 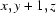; (iii) 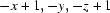.

**Fig. 1.**
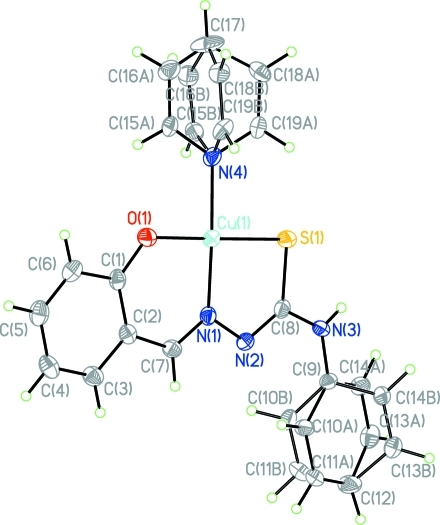
View of the molecular structure of the title compound. Displacement ellipsoids are drawn at the 50% probability level. Both positions of the disordered phenyl and pyridine rings are shown.

**Fig. 2.**
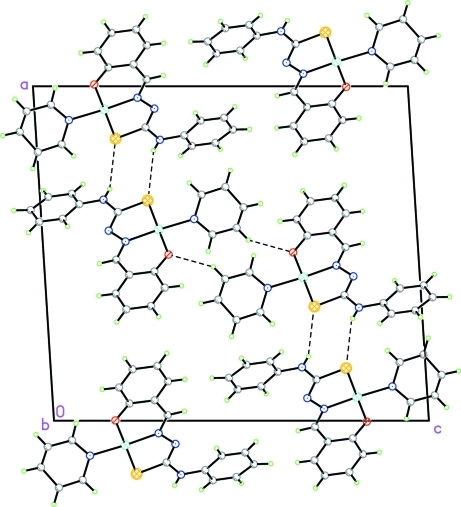
Crystal packing of title compound showing only the A position for the disordered phenyl and pyridine rings in a projection along the b axis. Dashed lines indicate hydrogen bonds.

## supplementary crystallographic information

### Comment

Thiosemicarbazones and their metal complexes attract constant scientific interest due to their antimicrobial, antifungal and antitumoral activities (Garoufilis *et al.*, 2009; Stanojkovic *et al.*, 2010). Moreover, some thiosemicarbazones are used as reagents for determination of Co(II), Ni(II), Cu(II) and Pd(II) by solid phase microextraction in HPLC (Kaur *et al.*, 2007). Several crystal structures of salicylaldehyde (4)-phenylthiosemicarbazone metal complexes with Ni(II), Cu(II), Co(II) and Zn(II) have been reported previously (John *et al.*, 2002; Naik *et al.*, 2003; Cao *et al.*, 2007; Seena & Kurup, 2008).

The title compound crystallizes with one molecule in the asymmetric unit (Fig. 1), which differs from the previously reported Ni compound (Cao *et al.*, 2007) that crystallizes with two different molecules. The copper atom in the title structure exhibits a slightly distorted square-planar coordination with a mean deviation from the Cu1/O1/N1/S1/N4 plane of 0.0485 Å. The doubly deprotonated ligand molecule coordinates to the copper(II) atom in a tridentate manner *via* a phenyl oxygen, an azomethine nitrogen and a thioamide sulfur atom, creating five- and six-membered chelate metalla rings. The pyridine molecule completes the square-planar coordination environment of the Cu(II) atom. Values of Cu—O, Cu—N and Cu—S bond lengths are in a good agreement with related structures (Naik *et al.*, 2003). The C8—S1 (1.749 (3) Å) and C8—N2 (1.299 (3) Å) bond lengths indicate the presence of the thiol tautomeric form of the thiosemicarbazone in the structure. The value of the torsion angle N1—N2—C8—N3 = -179.9 (2)° confirms the presence of the *E*-isomer for the coordinating ligand molecule.

The title structure contains several planar fragments. The major part Cu1/O1/N1/N2/S1/C1—C9 (denoted as plane A) has a mean deviation from the least squares plane of 0.023 Å. The disordered phenyl ring contains two planar fragments C9/C10A/C11A/C12/C13A/C14A (plane B) and C9/C10B/C11B/C12/C13B/C14B (plane C) with a mean deviation from the corresponding least squares planes of 0.026 and 0.020 Å, respectively. The dihedral angles between planes A/B, A/C and B/C are 17.00 (19)°, 26.3 (2)° and 43.1 (3)°. The mean deviations from the least squares planes for the disordered pyridine ring N4/C15A/C16A/C17/C18A/C19A (plane D) and N4/C15B/C16B/C17/C18B/C19B (plane E) amount  to 0.0054 and 0.0329 Å, respectively, with a dihedral angle between planes D and E of 51.2 (3)°.

The crystal packing of the title compound (Fig. 2) is characterised by an alternating arrangement of disordered phenyl and pyridine rings in neighboring molecules (configuration A and B). An extensive network of weak N—H···S, C—H···O, C—H···N and C—H···S hydrogen bonding interactions additionally stabilizes the crystal structure (Table 2).

### Experimental

20 ml (5x10^-3^ M) of an aqueous solution of copper acetate was stirred in a cone flask for 2 hours with a mixture that contained 10 ml (10^-2^ M) of an ethanolic solution of salicylaldehyde (4)-phenylthiosemicarbazone and 2 ml of pyridine. The resulting solution was left for 3 days in a dark place. As a result, brown needle-like crystals of title compound were isolated from the solution.

### Refinement

The structure refinement indicates rotational disorder of phenyl (C(9)—C(14)) and pyridine (C(15)—C19)N(4)) rings. For this reason, both positions for the atoms C(10), C(11), C(12), C(13), C(15), C(16), C(18) and C(19) were refined with occupancies of 0.5. All disordered ring atoms were refined anisotropically. The hydrogen atom bonded to N(3) was found from a difference Fourier map and was refined freely. All other hydrogen atoms were constrained geometrically and refined using a "riding model" on the parent atom with d(C—H) = 0.95 Å and Uiso(H) = 1.5Ueq(C).

### Crystal data

**Table:**

[Cu(C_14_H_11_N_3_OS)(C_5_H_5_N)]	*F*(000) = 844
*M**_r_* = 411.96	*D*_x_ = 1.605 Mg m^−^^3^
Monoclinic, *P*2_1_/*n*	Mo *K*α radiation, λ = 0.71073 Å
Hall symbol: -P 2yn	Cell parameters from 2588 reflections
*a* = 18.2958 (17) Å	θ = 2.2–23.9°
*b* = 4.5610 (5) Å	µ = 1.42 mm^−^^1^
*c* = 20.473 (2) Å	*T* = 173 K
β = 93.602 (7)°	Needle, brown
*V* = 1705.1 (3) Å^3^	0.50 × 0.06 × 0.05 mm
*Z* = 4	

### Data collection

**Table:**

Bruker APEXII CCD diffractometer	3493 independent reflections
Radiation source: fine-focus sealed tube	2522 reflections with *I* > 2σ(*I*)
graphite	*R*_int_ = 0.078
Detector resolution: 8.33 pixels mm^-1^	θ_max_ = 26.4°, θ_min_ = 1.5°
φ and ω scans	*h* = −22→22
Absorption correction: multi-scan (*SADABS*; Bruker, 2005)	*k* = −5→5
*T*_min_ = 0.540, *T*_max_ = 0.938	*l* = −25→25
19901 measured reflections	

### Refinement

**Table:**

Refinement on *F*^2^	Primary atom site location: structure-invariant direct methods
Least-squares matrix: full	Secondary atom site location: difference Fourier map
*R*[*F*^2^ > 2σ(*F*^2^)] = 0.038	Hydrogen site location: inferred from neighbouring sites
*wR*(*F*^2^) = 0.080	H atoms treated by a mixture of independent and constrained refinement
*S* = 1.02	*w* = 1/[σ^2^(*F*_o_^2^) + (0.0237*P*)^2^ + 1.1058*P*] where *P* = (*F*_o_^2^ + 2*F*_c_^2^)/3
3493 reflections	(Δ/σ)_max_ = 0.001
311 parameters	Δρ_max_ = 0.33 e Å^−^^3^
0 restraints	Δρ_min_ = −0.45 e Å^−^^3^

### Special details

**Table:**

Geometry. All esds (except the esd in the dihedral angle between two l.s. planes) are estimated using the full covariance matrix. The cell esds are taken into account individually in the estimation of esds in distances, angles and torsion angles; correlations between esds in cell parameters are only used when they are defined by crystal symmetry. An approximate (isotropic) treatment of cell esds is used for estimating esds involving l.s. planes.
Refinement. Refinement of *F*^2^ against ALL reflections. The weighted *R*-factor wR and goodness of fit *S* are based on *F*^2^, conventional *R*-factors *R* are based on *F*, with *F* set to zero for negative *F*^2^. The threshold expression of *F*^2^ > σ(*F*^2^) is used only for calculating *R*-factors(gt) etc. and is not relevant to the choice of reflections for refinement. *R*-factors based on *F*^2^ are statistically about twice as large as those based on *F*, and *R*- factors based on ALL data will be even larger.

### Fractional atomic coordinates and isotropic or equivalent isotropic displacement parameters (Å^2^)

**Table:**

	*x*	*y*	*z*	*U*_iso_*/*U*_eq_	Occ. (<1)
Cu1	0.428933 (18)	0.04089 (8)	0.687460 (17)	0.02260 (12)	
S1	0.34247 (4)	0.37030 (18)	0.71230 (4)	0.0274 (2)	
N1	0.46424 (12)	0.0759 (5)	0.77777 (11)	0.0194 (5)	
N2	0.43213 (12)	0.2699 (5)	0.82056 (11)	0.0207 (6)	
N3	0.33957 (14)	0.6080 (6)	0.82989 (13)	0.0224 (6)	
H3N	0.3077 (15)	0.674 (6)	0.8115 (14)	0.017 (9)*	
N4	0.39751 (13)	0.0530 (5)	0.59166 (11)	0.0223 (6)	
O1	0.50312 (10)	−0.2308 (4)	0.66541 (9)	0.0253 (5)	
C1	0.55353 (15)	−0.3487 (6)	0.70570 (15)	0.0217 (7)	
C2	0.56305 (14)	−0.2790 (6)	0.77319 (14)	0.0200 (7)	
C3	0.61943 (15)	−0.4170 (6)	0.81158 (15)	0.0251 (7)	
H3A	0.6257	−0.3696	0.8568	0.030*	
C4	0.66565 (16)	−0.6175 (7)	0.78620 (16)	0.0288 (8)	
H4A	0.7035	−0.7072	0.8131	0.035*	
C5	0.65588 (16)	−0.6865 (7)	0.72001 (16)	0.0306 (8)	
H5A	0.6874	−0.8251	0.7016	0.037*	
C6	0.60151 (16)	−0.5578 (7)	0.68106 (15)	0.0270 (7)	
H6A	0.5959	−0.6106	0.6361	0.032*	
C7	0.51859 (15)	−0.0705 (6)	0.80498 (14)	0.0214 (7)	
H7A	0.5301	−0.0368	0.8503	0.026*	
C8	0.37630 (15)	0.4103 (6)	0.79364 (14)	0.0203 (7)	
C9	0.35281 (15)	0.6925 (6)	0.89537 (13)	0.0187 (6)	
C12	0.37031 (17)	0.8705 (7)	1.02567 (15)	0.0313 (8)	
H12A	0.3713	0.9194	1.0708	0.038*	
C17	0.3627 (2)	0.0996 (7)	0.45900 (16)	0.0404 (9)	
H17A	0.3515	0.1147	0.4132	0.048*	
C10A	0.3965 (3)	0.5278 (16)	0.9414 (3)	0.0229 (15)	0.50
H10A	0.4190	0.3501	0.9292	0.027*	0.50
C11A	0.4055 (4)	0.633 (2)	1.0048 (4)	0.029 (2)	0.50
H11A	0.4380	0.5320	1.0350	0.035*	0.50
C13A	0.3313 (3)	1.0474 (15)	0.9773 (3)	0.0267 (14)	0.50
H13A	0.3120	1.2319	0.9892	0.032*	0.50
C14A	0.3216 (3)	0.9506 (15)	0.9135 (3)	0.0238 (14)	0.50
H14A	0.2933	1.0630	0.8821	0.029*	0.50
C15A	0.4434 (3)	0.1199 (15)	0.5460 (3)	0.0262 (15)	0.50
H15A	0.4932	0.1536	0.5598	0.031*	0.50
C16A	0.4238 (4)	0.1436 (16)	0.4804 (3)	0.0291 (16)	0.50
H16A	0.4599	0.1967	0.4513	0.035*	0.50
C18A	0.3023 (3)	0.0198 (13)	0.5036 (3)	0.0263 (14)	0.50
H18A	0.2530	−0.0121	0.4879	0.032*	0.50
C19A	0.3251 (3)	−0.0019 (13)	0.5681 (3)	0.0226 (14)	0.50
H19A	0.2904	−0.0568	0.5985	0.027*	0.50
C10B	0.4197 (4)	0.6784 (15)	0.9301 (3)	0.0228 (15)	0.50
H10B	0.4615	0.6127	0.9090	0.027*	0.50
C11B	0.4269 (5)	0.7590 (17)	0.9955 (4)	0.0270 (19)	0.50
H11B	0.4727	0.7347	1.0194	0.032*	0.50
C13B	0.2999 (3)	0.8776 (14)	0.9931 (3)	0.0250 (14)	0.50
H13B	0.2583	0.9315	1.0160	0.030*	0.50
C14B	0.2924 (3)	0.8055 (14)	0.9280 (3)	0.0226 (14)	0.50
H14B	0.2464	0.8313	0.9045	0.027*	0.50
C15B	0.4194 (3)	0.2973 (15)	0.5609 (3)	0.0230 (14)	0.50
H15B	0.4436	0.4488	0.5857	0.028*	0.50
C16B	0.4073 (3)	0.3304 (16)	0.4944 (3)	0.0259 (15)	0.50
H16B	0.4265	0.4928	0.4720	0.031*	0.50
C18B	0.3501 (3)	−0.1385 (13)	0.4924 (3)	0.0241 (14)	0.50
H18B	0.3295	−0.3044	0.4701	0.029*	0.50
C19B	0.3662 (3)	−0.1547 (14)	0.5595 (3)	0.0258 (15)	0.50
H19B	0.3533	−0.3272	0.5821	0.031*	0.50

### Atomic displacement parameters (Å^2^)

**Table:**

	*U*^11^	*U*^22^	*U*^33^	*U*^12^	*U*^13^	*U*^23^
Cu1	0.02066 (19)	0.0297 (2)	0.0176 (2)	0.00297 (17)	0.00239 (14)	−0.00190 (17)
S1	0.0248 (4)	0.0390 (5)	0.0181 (4)	0.0082 (4)	−0.0003 (3)	−0.0037 (4)
N1	0.0165 (12)	0.0212 (13)	0.0207 (14)	0.0006 (11)	0.0025 (10)	−0.0039 (11)
N2	0.0205 (13)	0.0245 (14)	0.0173 (14)	0.0026 (11)	0.0024 (11)	−0.0042 (11)
N3	0.0187 (14)	0.0313 (16)	0.0166 (14)	0.0067 (12)	−0.0022 (12)	−0.0006 (12)
N4	0.0238 (13)	0.0227 (14)	0.0208 (14)	0.0049 (12)	0.0055 (11)	−0.0011 (12)
O1	0.0236 (11)	0.0313 (12)	0.0208 (11)	0.0048 (10)	−0.0005 (9)	−0.0031 (10)
C1	0.0172 (15)	0.0207 (16)	0.0276 (18)	−0.0021 (13)	0.0043 (13)	0.0027 (14)
C2	0.0184 (15)	0.0165 (15)	0.0252 (17)	−0.0038 (12)	0.0017 (13)	0.0027 (13)
C3	0.0254 (16)	0.0245 (17)	0.0251 (17)	0.0002 (14)	−0.0015 (13)	−0.0031 (14)
C4	0.0231 (16)	0.0250 (18)	0.038 (2)	0.0038 (14)	−0.0031 (15)	0.0021 (15)
C5	0.0280 (17)	0.0256 (18)	0.039 (2)	0.0064 (15)	0.0084 (16)	−0.0021 (16)
C6	0.0297 (17)	0.0267 (17)	0.0255 (17)	0.0006 (14)	0.0081 (14)	−0.0019 (14)
C7	0.0213 (15)	0.0228 (16)	0.0198 (16)	−0.0038 (13)	−0.0010 (13)	−0.0040 (13)
C8	0.0177 (15)	0.0228 (17)	0.0208 (16)	−0.0019 (13)	0.0049 (12)	−0.0010 (13)
C9	0.0204 (15)	0.0203 (16)	0.0154 (16)	−0.0019 (13)	0.0017 (12)	−0.0016 (13)
C12	0.0372 (19)	0.036 (2)	0.0198 (17)	0.0057 (16)	−0.0021 (15)	−0.0090 (15)
C17	0.078 (3)	0.028 (2)	0.0147 (18)	0.014 (2)	0.0007 (19)	−0.0043 (15)
C10A	0.026 (4)	0.025 (4)	0.017 (4)	0.007 (3)	−0.001 (3)	−0.001 (3)
C11A	0.020 (4)	0.047 (6)	0.019 (4)	0.005 (4)	−0.003 (3)	0.000 (4)
C13A	0.028 (3)	0.021 (3)	0.032 (4)	0.000 (3)	0.007 (3)	−0.008 (3)
C14A	0.025 (3)	0.025 (4)	0.022 (4)	−0.001 (3)	0.002 (3)	0.003 (3)
C15A	0.020 (3)	0.036 (4)	0.023 (4)	0.007 (3)	0.006 (3)	−0.006 (3)
C16A	0.036 (4)	0.028 (4)	0.024 (4)	0.007 (3)	0.007 (3)	0.006 (3)
C18A	0.025 (3)	0.023 (4)	0.029 (4)	0.004 (3)	−0.009 (3)	−0.006 (3)
C19A	0.019 (3)	0.021 (4)	0.028 (4)	0.000 (3)	0.007 (3)	−0.001 (3)
C10B	0.023 (4)	0.022 (4)	0.025 (4)	0.004 (3)	0.009 (3)	0.002 (3)
C11B	0.024 (5)	0.029 (5)	0.028 (5)	0.002 (3)	−0.009 (4)	0.000 (4)
C13B	0.024 (3)	0.025 (4)	0.026 (4)	0.002 (3)	0.006 (3)	−0.002 (3)
C14B	0.020 (3)	0.026 (4)	0.022 (4)	0.006 (3)	0.002 (3)	0.002 (3)
C15B	0.020 (3)	0.026 (4)	0.023 (4)	0.002 (3)	−0.001 (3)	−0.007 (3)
C16B	0.026 (4)	0.024 (4)	0.028 (4)	0.002 (3)	0.002 (3)	0.006 (3)
C18B	0.027 (3)	0.020 (3)	0.025 (4)	−0.003 (3)	0.002 (3)	−0.009 (3)
C19B	0.030 (4)	0.024 (4)	0.024 (4)	−0.004 (3)	0.011 (3)	−0.002 (3)

### Geometric parameters (Å, °)

**Table:**

Cu1—O1	1.9126 (19)	C12—C11A	1.344 (10)
Cu1—N1	1.926 (2)	C12—C13B	1.414 (7)
Cu1—N4	2.010 (2)	C12—C13A	1.433 (7)
Cu1—S1	2.2626 (8)	C12—H12A	0.9500
S1—C8	1.749 (3)	C17—C16A	1.191 (7)
N1—C7	1.294 (3)	C17—C18B	1.311 (7)
N1—N2	1.400 (3)	C17—C16B	1.491 (8)
N2—C8	1.299 (3)	C17—C18A	1.522 (7)
N3—C8	1.370 (4)	C17—H17A	0.9500
N3—C9	1.401 (4)	C10A—C11A	1.383 (10)
N3—H3N	0.74 (3)	C10A—H10A	0.9500
N4—C19B	1.270 (6)	C11A—H11A	0.9500
N4—C15A	1.330 (6)	C13A—C14A	1.380 (8)
N4—C15B	1.353 (7)	C13A—H13A	0.9500
N4—C19A	1.403 (6)	C14A—H14A	0.9500
O1—C1	1.313 (3)	C15A—C16A	1.373 (9)
C1—C6	1.411 (4)	C15A—H15A	0.9500
C1—C2	1.418 (4)	C16A—H16A	0.9500
C2—C3	1.406 (4)	C18A—C19A	1.363 (8)
C2—C7	1.434 (4)	C18A—H18A	0.9500
C3—C4	1.370 (4)	C19A—H19A	0.9500
C3—H3A	0.9500	C10B—C11B	1.387 (11)
C4—C5	1.392 (4)	C10B—H10B	0.9500
C4—H4A	0.9500	C11B—H11B	0.9500
C5—C6	1.368 (4)	C13B—C14B	1.371 (8)
C5—H5A	0.9500	C13B—H13B	0.9500
C6—H6A	0.9500	C14B—H14B	0.9500
C7—H7A	0.9500	C15B—C16B	1.374 (8)
C9—C14A	1.369 (7)	C15B—H15B	0.9500
C9—C10B	1.379 (7)	C16B—H16B	0.9500
C9—C10A	1.413 (7)	C18B—C19B	1.388 (8)
C9—C14B	1.424 (6)	C18B—H18B	0.9500
C12—C11B	1.338 (9)	C19B—H19B	0.9500
			
O1—Cu1—N1	94.59 (9)	C18B—C17—C16B	116.1 (4)
O1—Cu1—N4	87.20 (9)	C16A—C17—C18A	121.2 (5)
N1—Cu1—N4	172.99 (10)	C16A—C17—H17A	119.4
O1—Cu1—S1	178.70 (7)	C18B—C17—H17A	122.7
N1—Cu1—S1	85.77 (7)	C16B—C17—H17A	120.4
N4—Cu1—S1	92.29 (7)	C18A—C17—H17A	119.4
C8—S1—Cu1	94.17 (10)	C11A—C10A—C9	118.1 (6)
C7—N1—N2	113.4 (2)	C11A—C10A—H10A	121.0
C7—N1—Cu1	124.9 (2)	C9—C10A—H10A	121.0
N2—N1—Cu1	121.63 (17)	C12—C11A—C10A	123.1 (7)
C8—N2—N1	113.3 (2)	C12—C11A—H11A	118.4
C8—N3—C9	129.6 (3)	C10A—C11A—H11A	118.4
C8—N3—H3N	113 (2)	C14A—C13A—C12	120.2 (5)
C9—N3—H3N	117 (2)	C14A—C13A—H13A	119.9
C19B—N4—C15B	120.6 (4)	C12—C13A—H13A	119.9
C15A—N4—C19A	115.0 (4)	C9—C14A—C13A	120.1 (6)
C19B—N4—Cu1	125.3 (3)	C9—C14A—H14A	120.0
C15A—N4—Cu1	122.5 (3)	C13A—C14A—H14A	120.0
C15B—N4—Cu1	113.7 (3)	N4—C15A—C16A	124.6 (6)
C19A—N4—Cu1	122.4 (3)	N4—C15A—H15A	117.7
C1—O1—Cu1	126.73 (18)	C16A—C15A—H15A	117.7
O1—C1—C6	118.7 (3)	C17—C16A—C15A	121.9 (6)
O1—C1—C2	124.0 (3)	C17—C16A—H16A	119.1
C6—C1—C2	117.3 (3)	C15A—C16A—H16A	119.1
C3—C2—C1	119.0 (3)	C19A—C18A—C17	114.2 (5)
C3—C2—C7	117.4 (3)	C19A—C18A—H18A	122.9
C1—C2—C7	123.5 (3)	C17—C18A—H18A	122.9
C4—C3—C2	122.4 (3)	C18A—C19A—N4	123.0 (5)
C4—C3—H3A	118.8	C18A—C19A—H19A	118.5
C2—C3—H3A	118.8	N4—C19A—H19A	118.5
C3—C4—C5	118.4 (3)	C9—C10B—C11B	120.8 (6)
C3—C4—H4A	120.8	C9—C10B—H10B	119.6
C5—C4—H4A	120.8	C11B—C10B—H10B	119.6
C6—C5—C4	121.0 (3)	C12—C11B—C10B	121.2 (7)
C6—C5—H5A	119.5	C12—C11B—H11B	119.4
C4—C5—H5A	119.5	C10B—C11B—H11B	119.4
C5—C6—C1	121.9 (3)	C14B—C13B—C12	119.0 (5)
C5—C6—H6A	119.1	C14B—C13B—H13B	120.5
C1—C6—H6A	119.1	C12—C13B—H13B	120.5
N1—C7—C2	126.1 (3)	C13B—C14B—C9	120.7 (5)
N1—C7—H7A	117.0	C13B—C14B—H14B	119.7
C2—C7—H7A	117.0	C9—C14B—H14B	119.7
N2—C8—N3	119.6 (3)	N4—C15B—C16B	121.2 (6)
N2—C8—S1	125.1 (2)	N4—C15B—H15B	119.4
N3—C8—S1	115.3 (2)	C16B—C15B—H15B	119.4
C14A—C9—N3	116.5 (4)	C15B—C16B—C17	116.9 (5)
C10B—C9—N3	125.1 (4)	C15B—C16B—H16B	121.5
C14A—C9—C10A	120.2 (4)	C17—C16B—H16B	121.5
N3—C9—C10A	123.3 (4)	C17—C18B—C19B	121.8 (5)
C10B—C9—C14B	117.7 (4)	C17—C18B—H18B	119.1
N3—C9—C14B	117.1 (3)	C19B—C18B—H18B	119.1
C11B—C12—C13B	120.0 (5)	N4—C19B—C18B	122.3 (6)
C11A—C12—C13A	117.5 (5)	N4—C19B—H19B	118.8
C11A—C12—H12A	121.2	C18B—C19B—H19B	118.8
C13A—C12—H12A	121.2		
			
N1—Cu1—S1—C8	1.70 (11)	C13B—C12—C13A—C14A	−77.5 (7)
N4—Cu1—S1—C8	−171.55 (12)	C10B—C9—C14A—C13A	−36.8 (7)
O1—Cu1—N1—C7	−3.4 (2)	N3—C9—C14A—C13A	179.2 (5)
S1—Cu1—N1—C7	177.9 (2)	C10A—C9—C14A—C13A	−1.6 (8)
O1—Cu1—N1—N2	176.30 (19)	C14B—C9—C14A—C13A	78.3 (8)
S1—Cu1—N1—N2	−2.45 (18)	C12—C13A—C14A—C9	−3.7 (9)
C7—N1—N2—C8	−178.1 (2)	C19B—N4—C15A—C16A	44.8 (8)
Cu1—N1—N2—C8	2.2 (3)	C15B—N4—C15A—C16A	−86.2 (8)
O1—Cu1—N4—C19B	70.3 (4)	C19A—N4—C15A—C16A	1.6 (9)
S1—Cu1—N4—C19B	−110.8 (4)	Cu1—N4—C15A—C16A	−177.0 (5)
O1—Cu1—N4—C15A	−55.3 (4)	C18B—C17—C16A—C15A	−44.0 (8)
S1—Cu1—N4—C15A	123.5 (4)	C16B—C17—C16A—C15A	77.8 (8)
O1—Cu1—N4—C15B	−103.2 (3)	C18A—C17—C16A—C15A	1.2 (10)
S1—Cu1—N4—C15B	75.7 (3)	N4—C15A—C16A—C17	−1.4 (12)
O1—Cu1—N4—C19A	126.2 (3)	C16A—C17—C18A—C19A	−1.4 (8)
S1—Cu1—N4—C19A	−55.0 (3)	C18B—C17—C18A—C19A	69.4 (6)
N1—Cu1—O1—C1	4.4 (2)	C16B—C17—C18A—C19A	−41.7 (6)
N4—Cu1—O1—C1	177.6 (2)	C17—C18A—C19A—N4	1.8 (8)
Cu1—O1—C1—C6	176.65 (19)	C19B—N4—C19A—C18A	−73.6 (7)
Cu1—O1—C1—C2	−3.4 (4)	C15A—N4—C19A—C18A	−2.0 (8)
O1—C1—C2—C3	−179.4 (3)	C15B—N4—C19A—C18A	43.1 (7)
C6—C1—C2—C3	0.6 (4)	Cu1—N4—C19A—C18A	176.6 (4)
O1—C1—C2—C7	−0.2 (4)	C14A—C9—C10B—C11B	42.1 (8)
C6—C1—C2—C7	179.8 (3)	N3—C9—C10B—C11B	−178.0 (5)
C1—C2—C3—C4	−0.1 (4)	C10A—C9—C10B—C11B	−78.4 (9)
C7—C2—C3—C4	−179.3 (3)	C14B—C9—C10B—C11B	2.9 (9)
C2—C3—C4—C5	−0.3 (4)	C11A—C12—C11B—C10B	82.0 (13)
C3—C4—C5—C6	0.0 (5)	C13B—C12—C11B—C10B	7.1 (10)
C4—C5—C6—C1	0.6 (5)	C13A—C12—C11B—C10B	−36.3 (8)
O1—C1—C6—C5	179.1 (3)	C9—C10B—C11B—C12	−4.4 (11)
C2—C1—C6—C5	−0.9 (4)	C11B—C12—C13B—C14B	−8.5 (9)
N2—N1—C7—C2	−178.4 (2)	C11A—C12—C13B—C14B	−40.6 (8)
Cu1—N1—C7—C2	1.3 (4)	C13A—C12—C13B—C14B	70.9 (7)
C3—C2—C7—N1	−179.6 (3)	C12—C13B—C14B—C9	7.3 (9)
C1—C2—C7—N1	1.2 (4)	C14A—C9—C14B—C13B	−84.6 (8)
N1—N2—C8—N3	−179.9 (2)	C10B—C9—C14B—C13B	−4.6 (8)
N1—N2—C8—S1	−0.2 (3)	N3—C9—C14B—C13B	176.3 (5)
C9—N3—C8—N2	0.5 (5)	C10A—C9—C14B—C13B	32.3 (8)
C9—N3—C8—S1	−179.3 (2)	C19B—N4—C15B—C16B	1.2 (8)
Cu1—S1—C8—N2	−1.4 (3)	C15A—N4—C15B—C16B	62.1 (7)
Cu1—S1—C8—N3	178.4 (2)	C19A—N4—C15B—C16B	−46.9 (7)
C8—N3—C9—C14A	161.8 (4)	Cu1—N4—C15B—C16B	175.0 (5)
C8—N3—C9—C10B	25.5 (6)	N4—C15B—C16B—C17	5.9 (9)
C8—N3—C9—C10A	−17.5 (6)	C16A—C17—C16B—C15B	−81.2 (8)
C8—N3—C9—C14B	−155.4 (4)	C18B—C17—C16B—C15B	−11.6 (8)
C14A—C9—C10A—C11A	1.0 (9)	C18A—C17—C16B—C15B	38.9 (7)
C10B—C9—C10A—C11A	74.9 (9)	C16A—C17—C18B—C19B	48.0 (7)
N3—C9—C10A—C11A	−179.8 (5)	C16B—C17—C18B—C19B	10.8 (8)
C14B—C9—C10A—C11A	−38.5 (8)	C18A—C17—C18B—C19B	−77.4 (6)
C11B—C12—C11A—C10A	−83.6 (13)	C15A—N4—C19B—C18B	−39.3 (7)
C13B—C12—C11A—C10A	34.8 (9)	C15B—N4—C19B—C18B	−2.7 (8)
C13A—C12—C11A—C10A	−10.2 (10)	C19A—N4—C19B—C18B	81.0 (7)
C9—C10A—C11A—C12	5.2 (11)	Cu1—N4—C19B—C18B	−175.8 (4)
C11B—C12—C13A—C14A	40.7 (8)	C17—C18B—C19B—N4	−3.9 (9)
C11A—C12—C13A—C14A	9.4 (8)		

### Hydrogen-bond geometry (Å, °)

**Table:**

*D*—H···*A*	*D*—H	H···*A*	*D*···*A*	*D*—H···*A*
N3—H3N···S1^i^	0.74 (3)	2.90 (3)	3.593 (3)	157 (3)
C10A—H10A···N2	0.95	2.28	2.852 (7)	118
C10B—H10B···N2	0.95	2.43	2.936 (7)	113
C14B—H14B···S1^i^	0.95	2.81	3.679 (6)	152
C15B—H15B···O1^ii^	0.95	2.40	3.339 (7)	169
C16A—H16A···O1^iii^	0.95	2.53	3.371 (7)	148

Symmetry codes: (i) −*x*+1/2, *y*+1/2, −*z*+3/2; (ii) *x*, *y*+1, *z*; (iii) −*x*+1, −*y*, −*z*+1.
